# Jerusalem

**Published:** 1871-10

**Authors:** 


### JERUSALEM.

I WENT to meeting last Sunday night, and heard them talking about an old maid in London, who, like a good many old maids, was doing good in her day and generation, for she had a large heart, a deep pocket, and a great deal of money in it; in fact, she is such a good "business man," that she has been making money all her life, and is making money still, although she is all the time finding out ways of spending it to advantage, — not her own, except so far as making others happy. One day she buys a church, and gives it to a poor parish not able to build one, just as some of New York's merchant princes have done — Stewart, Vanderbilt, Agnew, and others. Being worth her millions, she is able to do a great deal, not only at home but abroad. Among other things she heard that Jerusalem needed greatly to be made more cleanly, and to be better supplied with water; so she sent some men over there, and had it done. Then it was suggested that Solomon's Temple must be somewhere, at least its foundations, and that most likely it was covered over a hundred feet or more with the offal and rubbish of a city in the course of ages; this led to digging a hole ever so deep, down perpendicular; at the bottom of it they dug another hole sideways — several of them, in different directions; these were called passages, and, wonderful to tell, they came to a passage already dug, crawled through it on their stomachs, and came to an open space with apartments and arches, in such a way, that they have reason to suppose that these arches supported the temple, and that the passage through which they crept on their way to these arches was the drain through which the blood passed which came from the animals slaughtered in the temple service. Various articles have been found in these subterranean apartments, which possess considerable interest, and it is not improbable that others may be brought to the light of day, which have been covered up for two thousand years. Not long ago, while persons were digging in that country, some bright pieces were turned up, as large as a nickel cent. On examination they were found to be of pure gold; on further search, eight or ten thousand more were discovered, worth five dollars each; on one side was the head of Philip of Macedon; on the other, that of Alexander the Great. How came they there? Alexander's death was sudden. Some government official had these in his care, and on the emergency of the occasion buried them, as a method of greater safety, to be exhumed when affairs became settled; but in the troublesome times may have been killed or have forgotten the exact spot. Such a conjecture is not unreasonable.

We saw one of these specimens, and know of their existence. Who knows but other things may be found, of the make of thousands of years ago. May not even the ark of the covenant be found in some of those hidden, underground recesses of the temple? Who shall say that the tables of stone may not have been hidden by some faithful Israelite, when he found that the temple was about to be invaded by sacrilegious hands; there is no evidence that the ark was ever destroyed, or that the second tables of the law were ever broken; but it is known that both these were regarded with great veneration, and that thousands would risk their lives to preserve them.

Is there a single reader of these lines who does not wish to have these explorations continued, for the bare chance of such discoveries, or of others which might have some writings on them confirmatory of Scripture history and fact? But it costs money to do all this. Americans are not willing that the British nation should have all the glory of these discoveries, and so have organized a society here in New York, and want ten thousand dollars for the first year's operations. It would be a shame to let the princely New Yorkers furnish all the means. So the reader, who may have sufficient enthusiasm to lead him to part with his money to aid in prosecuting such an enterprise, and thus supplement the efforts of the noble hearted Miss Burdett Coutts, of London, can send his contribution, payable to the order of Wm. E. Dodge, New York city, himself one of Nature's noblemen, in his boundless benevolence, in his princely charities, and in his personal efforts for the promotion of all plans calculated to advance human happiness. And when Jerusalem is cleaned, abundantly supplied with water, with a railroad thirty miles long to the Mediterranean Sea at Joppa, where Peter "tarried many days with Simon a tanner," a man may make a summer trip from New York to the Holy City, go over the Jordan, and return to Gotham easily, within ten weeks; taking in his way Liverpool, London, Cologne, the Rhine, Heidelberg, Dresden, Munich, the Tyrol, Lombardy, the Alps, the Appenines, across the Mediterranean to Alexandria, Cairo, the Pyramids, Suez, Red Sea, Wilderness of Sin, Jerusalem, Joppa, home!

The time from New York to London is twelve days, to Brindisi on the Mediterranean four more, to Alexandria and Jerusalem four more, or twenty days from Gotham to the city of the Great King. With five hundred dollars apiece, two "good fellows," young and strong, starting from New York, with four months' time, could easily see the best half of the world. Many a business man, by such an excursion, many professional men also, would add years to a useful life, by reason of the recuperation afforded to the mental, moral, and physical system; and a great pity is it, that some such "let up" from intense application is not planned for every four or five years of men's lives.

